# Investigating the relationship between cognitive impairment and brain white matter tracts using diffusion tensor imaging in patients with prolactinoma

**DOI:** 10.1007/s40618-024-02442-y

**Published:** 2024-10-03

**Authors:** Mustafa Duru, Ahmet Numan Demir, Ahmet Oz, Osman Aykan Kargin, Ali Tarik Altunc, Oznur Demirel, Serdar Arslan, Osman Kizilkilic, Burc Cagri Poyraz, Pinar Kadioglu

**Affiliations:** 1 Department of Internal Medicine, Istanbul University-Cerrahpasa, Istanbul, Turkey; 2 Department of Endocrinology, Metabolism, and Diabetes, Istanbul University-Cerrahpasa, Fatih, Istanbul, 34098 Turkey; 3 Department of Radiology, Istanbul University-Cerrahpasa, Istanbul, Turkey; 4 Department of Psychiatry, Istanbul University-Cerrahpasa, Istanbul, Turkey; 5 Pituitary Center, Istanbul University-Cerrahpasa, Istanbul, Turkey

**Keywords:** Cognitive dysfunction, Diffusion tensor imaging, Prolactinoma, White matter tracts

## Abstract

**Background:**

Cognitive impairment is known to occur in patients with prolactinoma, but the underlying mechanism is unclear.

**Objective:**

To evaluate cognitive function in patients with prolactinoma and to investigate the basis of possible cognitive impairment in brain white matter changes using diffusion tensor imaging (DTI).

**Methods:**

37 consecutive patients with prolactinoma and 37 healthy controls of similar age, sex, and education were enrolled in the study. Hormone levels were determined in all participants, comprehensive neuropsychological testing was performed, and DTI was used to reconstruct and evaluate white matter tracts.

**Results:**

In patients with prolactinoma, short- and long-term visual and verbal memory, attention, concentration, and executive and language functions were impaired compared to the healthy group. When comparing the DTI results, lower fractional anisotropy (FA) values were found in the patients’ right uncinate fasciculus (R-UF), indicating neuronal damage. After applying the Bonferroni correction, the two groups had no significant difference in 42 tracts (*p* > 0.0012 for all). A positive correlation was found between poor FA scores on the R-UF and low scores on long-term memory, category and letter fluency tests. In addition, patients with hypoprolactinemia had the worst short-term memory scores, while normoprolactinemia had the best scores. Also, the poorer R-UF FA values were found in the patients with hypoprolactinemia and the highest in those with normoprolactinemia.

**Conclusion:**

This study is the first to investigate reasons for cognitive dysfunction in patients with prolactinoma by DTI. No significant structural changes were found in brain tracts of patients with prolactinoma. Still, there may be a link between potential damage in the R-UF and cognitive dysfunction, and further research is needed. In addition, the results showed that the development of hypoprolactinemia is associated with cognitive dysfunction and emphasized that overtreatment should be avoided.

**Supplementary Information:**

The online version contains supplementary material available at 10.1007/s40618-024-02442-y.

## Introduction

Prolactinoma is the most common functional pituitary adenoma, accounting for about half of all pituitary adenomas [1]. The main findings in women with prolactinoma are oligomenorrhea and galactorrhea, while in men with prolactinoma, they are impotence, infertility, and loss of libido [2]. Apart from its known effects on lactation and the gonadotropin axis, prolactin also affects many other systems. These include the metabolic, bones, and immune systems [3]. According to recent reports, prolactin overproduction also impairs cognitive function [4]. However, our knowledge of these effects and their mechanisms is limited. While hyperprolactinemia is known to have adverse effects, particularly on memory and attention, it is unclear whether hypoprolactinemia impairs cognitive function [5]. Various methods have been used to study the interaction of prolactin and cognitive function, including electroencephalography (EEG), magnetic resonance imaging (MRI), and morphometric measurements of brain volume [6, 7].

The integrity of brain tissue white matter can be assessed using recently available diffusion tensor imaging (DTI). This method quantitatively assesses the microstructure of the brain’s white matter [8]. No previous study has examined the structure of the brain white matter with DTI to understand the cause of cognitive dysfunction in patients with prolactinoma. In this study, cognitive function in patients with prolactinoma is assessed with neuropsychological tests, and the basis for possible cognitive impairment with DTI is investigated.

## Materials and methods

### Study design

This cross-sectional study was conducted in the endocrinology clinic of a tertiary care hospital. The Medical Research Ethics Committee of Istanbul University-Cerrahpasa approved the study.

### Participants

Patients treated for prolactinoma in our endocrinology clinic were consecutively included in the study. The inclusion criteria for the study were: (i) age between 18 and 65 years, (ii) definite diagnosis of prolactinoma [9], (iii) literacy, (iv) right-handedness. Exclusion criteria for the study were: (i) patients with secondary hyperprolactinemia such as pregnancy, primary hypothyroidism, treatment with antipsychotics, antidepressants and tranquilizers, renal and hepatic dysfunction, (ii) patients with known neurological and psychiatric disorders, iii) patients with a macroadenoma (≥ 1 cm) in the pituitary gland, iv) patients with hypopituitarism, v) patients who have undergone surgery or radiotherapy, vi) left-handed patients, vii) patients who do not consent to participate in the study.

For patients enrolled in the study, the prolactin level at the time of diagnosis, the size of the adenoma, the duration of treatment, the cumulative dose of cabergoline taken during treatment, and the current prolactin level were recorded. The change and duration of the patients’ prolactin levels during follow-up were recorded. Current prolactin levels during the study period were categorized as hypoprolactinemia (< 5 ng/ml), hyperprolactinemia (> 20 ng/ml), and normoprolactinemia (5–20 ng/ml) [9]. Patients were included in the study if their prolactin levels had not fluctuated for at least six months prior to the survey (i.e., hypo-, normal- or hyperprolactinemia). All patients underwent cranial MRI, and their cognitive abilities were assessed using neuropsychological tests. Age, sex, and level of education matched healthy control were included in the study. The control group was selected consecutively between January 2023 and September 2023 from individuals who had registered for a routine health check-up when applying for a job at our center and agreed to participate in the study. MRI of the brain, biochemical tests, and cognitive function tests were performed on all participants on the same day.

### Endocrinological definition

All patients with prolactinoma were treated as recommended in the guidelines [9]. In patients with hyperprolactinemia, other causes that could be secondary hyperprolactinemia were excluded. An MRI scan of the sella region was then performed. In the presence of an adenoma in the pituitary gland, a diagnosis of prolactinoma was made. In addition, hormone measurements were performed to confirm no hypersecretion of other anterior pituitary hormones. Adenomas were categorized according to their largest diameter into microadenomas (< 1 cm), macroadenomas (≥ 1 cm), and giant adenomas (≥ 4 cm). According to guideline recommendations, macroprolactin levels and the Hook effect were assessed in appropriate cases. Once a prolactinoma diagnosis was made, patient treatment was initiated according to the guidelines [9]. Drug treatment with dopamine agonists was used as the initial treatment. The guidelines’ recommendations gradually increased the dose until the prolactin level normalized. Surgery was only performed in selected cases with appropriate indications [9]. All patients were examined regularly with blood tests and, if necessary, an MRI of the sella. Patients whose symptoms disappeared, prolactin levels normalized, and pituitary adenoma did not enlarge were considered to be in remission (regardless of whether they were still taking medication). Patients who did not meet the remission criteria were considered to have active disease, and the dose of DA treatment was gradually increased until remission was achieved (up to the maximum tolerated DA dose). [9].

### Neuropsychological tests

The neuropsychological tests were carried out quietly while the participants were resting. The following tests were administered to all participants: Montreal Cognitive Assessment (MoCA) test, which provides a comprehensive cognitive assessment [10]; the Stroop test, which measures frontal attention and processing speed [11, 12]; Verbal Memory Processes Test [13]; Category and Letter Fluency Test, which assesses semantic memory, executive functions and language domain [14]; Wechsler Memory Scale (WMS) visual memory subtest, which assesses visual production [15]; the Beck Anxiety Scale [16, 17]; the Beck Depression Scale [18]. This comprehensive cognitive assessment took a total of approximately 1 h per patient.

### MRI acquisition and preprocessing

**Fig. 1:**
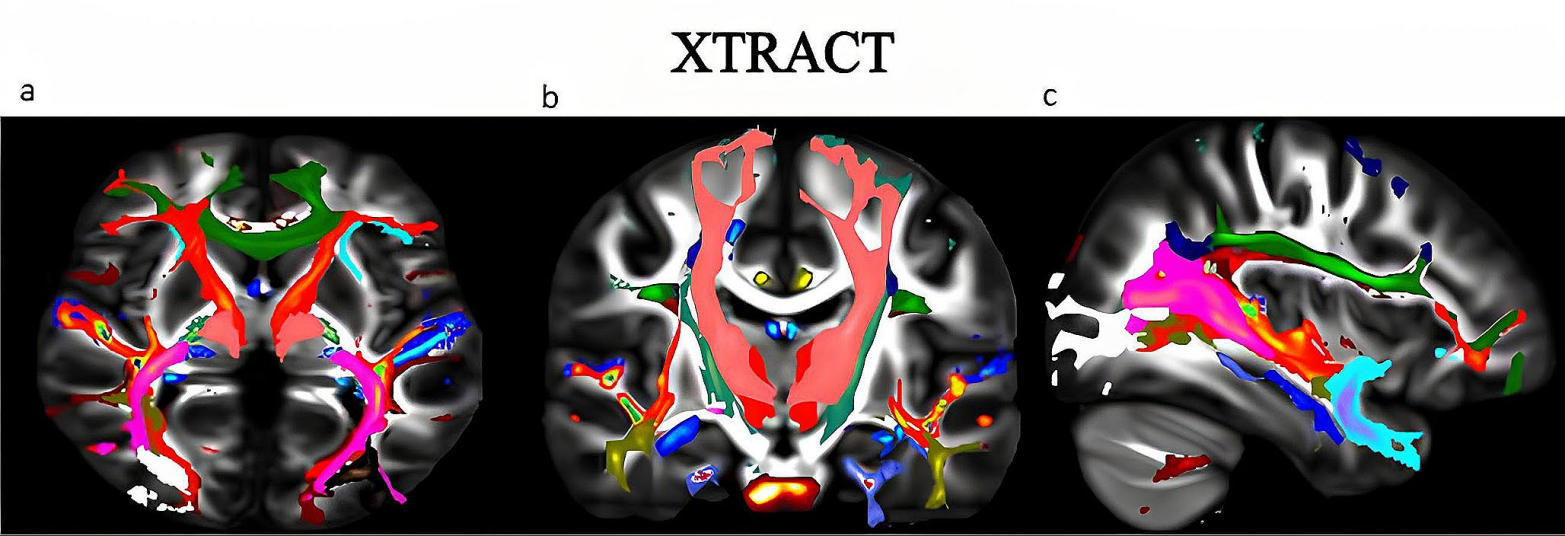
3-axis visualization of 42 white matter tracts obtained with the probabilistic tractography method of one participant from the control group using XTRACT on a high-resolution 3D T1 image **a**- Axial image, **b**- Coronal image, **c**- Sagittal image

All neuroimaging data were acquired using a 3T Philips Ingenia scanner (Philips, Best, the Netherlands) with a 32-channel head coil. The protocol comprises high-resolution 3D TFE (Turbo Field Echo), T1-weighted, and DTI sequences. 3D TFE T1-weighted imaging was obtained with FOV:256 × 256, TE:3.7 ms, TR:8.1 ms, flip angle:8° and 1 × 1 × 1 mm isotropic resolution. DTI is a single-shot, spin-echo, fat-suppressed echo-planar sequence with TR:2561 ms, TE:97 ms, FOV:256 × 256, 2 × 2 × 2 mm isotropic resolution, number of signal averages (NSA):1, b = 1000. It was obtained using s/mm2 and 64 non-collinear gradient direction parameters in the AP phase encoding direction. In addition, additional b = 0 s/mm2 images were acquired in the reverse phase-encoding direction to correct for DWI-induced susceptibility distortion artifacts. The total duration of the protocol is approximately 15 min. The FMRIB Software Library (FSL) v6.0 (The Analysis Group, FMRIB, Oxford, UK), available for academic purposes, was used to pre-process and analyze the acquired DTI data. Extra-brain structures were extracted from the Diffusion tensor images using BET (Brain Extraction Tool) [19, 20]. Correcting subject motion, EPI distortions, and eddy current artifacts in diffusion tensor images was performed using the top-up and Eddy tools [21, 22]. Fractional anisotropy (FA) maps were generated by calculating the diffusion tensor with DTIFIT on preprocessed images. Probabilistic tractography was carried out using GPU-based bedposts X. Using FSL’s XTRACT automated tractography software, 42 major white matter tracts in the brain (19 for both hemispheres and an additional four commissural tracts) were reconstructed, and the average FA value of each tract was calculated [23]. Figure 1 shows the 3-axis visualization of 42 white matter tracts obtained with the probabilistic tractography method of one participant from the control group using XTRACT on a high-resolution 3D T1 image.

### Hormonal examinations

Fasting blood samples were taken between 8:00 and 9:00 am. All hormone measurements were performed in the same laboratory using the same tests. An electrochemiluminescence immunoassay (ECLIA) was used to measure prolactin.

### Statistical analysis

Statistical analyses were performed using the Statistical Package for the Social Sciences (SPSS) software (version 21.0). Data were first analyzed for normality using the Kolmogorov–Smirnov test. Continuous variables were expressed as mean ± standard deviation (SD) and medians (interquartile range [IQR]). Student’s t-tests or analysis of variance (ANOVA) were used to compare means between groups with normal data distributions. Bonferroni correction was applied for multiple comparisons between groups. Medians were compared using the Mann–Whitney U and Kruskal-Wallis tests. Spearman’s rank order and Pearson correlation tests were used to calculate the correlation coefficients between continuous variables. Frequencies were compared using Pearson’s and Fisher’s exact tests. The results were evaluated at a 95% confidence interval, and a p-value < 0.05 was considered statistically significant.

## Results

### Participants characteristics

A total of 37 patients with prolactinoma and 37 healthy controls were included in the study. There were no differences between patients and controls regarding age, sex, working status, and education (*p* > 0.05 for all). A comparison of the sociodemographic characteristics of patients with prolactinoma and healthy controls can be found in Table 1. The initial median prolactin level of patients enrolled in the study was 90 [54–197] ng/ml. The prolactin level of the patients with prolactinoma was 11 [4–19] ng/ml at the time of examination, while it was 14 [11–17] ng/ml in the controls (*p* = 0.421). MRI examination of the patients revealed an adenoma size of 6 [4–9] mm. The patients’ median cumulative cabergoline dose during treatment was 96 [34–156] mg, and their weekly median cabergoline dose was 1.5 [1–2] mg/week. The treatment duration of the patients was 24 [18–44] months. Twenty-seven patients (73%) took cabergoline at the time of evaluation. Ten patients (27%) continued without medication, and at least two months or more had elapsed since discontinuation of the drug in the patients who had discontinued the medication. Distribution of patients according to prolactin level at the time of the study: 8 patients had hypoprolactinemia, 25 patients had normoprolactinemia, and four patients had hyperprolactinemia. The median duration of cabergoline use in patients with hypoprolactinemia was 15 [5.75–19.5] months, the median cumulative cabergoline dose was 98 [34–115.5] mg, and the weekly median cabergoline dose was 1.5 [1.25–1.75] mg/week. The median duration of cabergoline use in patients with normoprolactinemia was 6 [3–19.5] months, the median cumulative cabergoline dose was 78 [28–133] mg, and the weekly median cabergoline dose was 1.75 [1.5–2] mg/week. In patients with hyperprolactinemia, the median duration of cabergoline use was 22 [8–30] months, the median cumulative cabergoline dose was 143 [52–186] mg, and the weekly median cabergoline dose was 1.25 [1–1.5] mg/week. The duration of cabergoline use was significantly shorter in patients with normoprolactinemia than in other groups (*p* = 0.022). The cumulative cabergoline dose was significantly higher in patients with hyperprolactinemia than in the other groups (*p* = 0.018). The weekly cabergoline dose did not differ between the patient groups (*p* = 0.275).

**Table 1:** Comparison of the sociodemographic characteristics of participants’

Characteristics	Patients with Prolactinoma (*n* = 37)	Healthy Controls (*n* = 37)	*p*-values
Age, year, mean ± SD	30.1 ± 7.2	32.8 ± 7.1	0.119
Sex, male, n (%)	7 (19)	8 (22)	0.773
Working, yes, n (%)	30 (81)	30 (81)	1.0
Educational, n (%)			0.545
Primary School	14 (38)	16 (43)	
High School	8 (22)	9 (25)	
University	15 (40)	12 (33)	

SD, standard deviation

### Neuropsychological tests

A comparison of the results of the Verbal Memory Processes Test, the Category and Letter Fluency Test, the Stroop Test, the MoCA Test, and the WMS Test of the participants is shown in Table 2. The learning and long-term memory results were worse in patients with prolactinoma than in healthy controls (*p* < 0.005 for both). Patients with prolactinoma performed worse on category and letter fluency tests assessing attention, working memory, semantic memory, executive functions, and language domain (*p* < 0.001). In the MoCA test, which measures short-term memory as well as visuospatial abilities, executive functions, phonemic fluency, verbal abstraction, attention, concentration, working memory, language, and temporal and spatial orientation, the cognitive performance of patients with prolactinoma was reduced (*p* = 0.001). In the WMS, which tests visual memory, a decrease in visual memory function was found in patients with prolactinoma compared to healthy controls (*p* < 0.001). The results of the psychiatric screening tests administered to both groups are also shown in Table 3. It was found that the patients with prolactinoma were more anxious.

**Table 2:** Comparison of scores obtained from the participants’ verbal memory processes test, the Category and Letter fluency tests, the Stroop test, the MoCA test and the WMS test

Tests, mean ± SD	Patients with Prolactinoma (*n* = 37)	Healthy Controls (*n* = 37)	*p*-values*
Verbal Memory Processes Test			
Short-Term Memory Score	6.5 ± 1.6	7.8 ± 1.7	0.007
Learning Score	121.9 ± 13.8	131.2 ± 8.9	0.001
High Learning Score	13.8 ± 0.8	14.9 ± 0.4	0.022
Long-Term Memory Score	12.6 ± 1.6	13.9 ± 0.8	< 0.001
Total Memory Score	14.9 ± 0.3	14.9 ± 0.2	0.619
Category Test Score	20.6 ± 4.1	25.0 ± 4.2	< 0.001
Letter Fluency Test Score	28.9 ± 9.7	38.7 ± 10.7	< 0.001
Stroop Test Score	40.9 ± 10.6	36.4 ± 8.6	0.046
MoCA Test Score	24.2 ± 3.3	26.4 ± 2.5	0.001
WMS Total Score	12.1 ± 1.8	13.3 ± 0.8	< 0.001

MoCA, Montreal Cognitive Assessment Test; SD, standard deviation; WMS, Wechsler Memory Scale Visual Production Subtest

* The p values were obtained using a one-way analysis of covariance (ANCOVA) with the adjustment for age and sex covariance. Significance level when Bonferroni correction is applied: *p* < 0.05/10 = 0.005

Category Test, the number of animals counted in 1 min; Letter Fluency Test, the total number of words starting with the letters K, A, and S counted in one minute; Stroop Test; The difference in time between reading the color of the word and reading the word itself

**Table 3:** Comparison of participants’ psychiatric screening tests

Mood Tests	Patients with Prolactinoma (*n* = 37)	Healthy Controls (*n* = 37)	*p*-values
Beck Depression Score, mean ± SD	11.1 ± 9.0	7.7 ± 5.7	0.056
Beck Depression Category, n (%)			0.217
Minimal Depression	21 (56)	25 (68)	
Mild Depression	10 (27)	9 (24)	
Moderate Depression	5 (14)	3 (8)	
Severe Depression	1 (3)	0	
Beck Anxiety Score, mean ± SD	11.3 ± 4.3	6.8 ± 2.8	0.018
Beck Anxiety Category, n (%)			0.007
Normal	15 (40)	25 (68)	
Mild Anxiety	15 (40)	10 (27)	
Moderate Anxiety	3 (8)	2 (5)	
Severe Anxiety	4 (12)	0	

SD, standard deviation

### Radiological evaluation

**Fig. 2:**
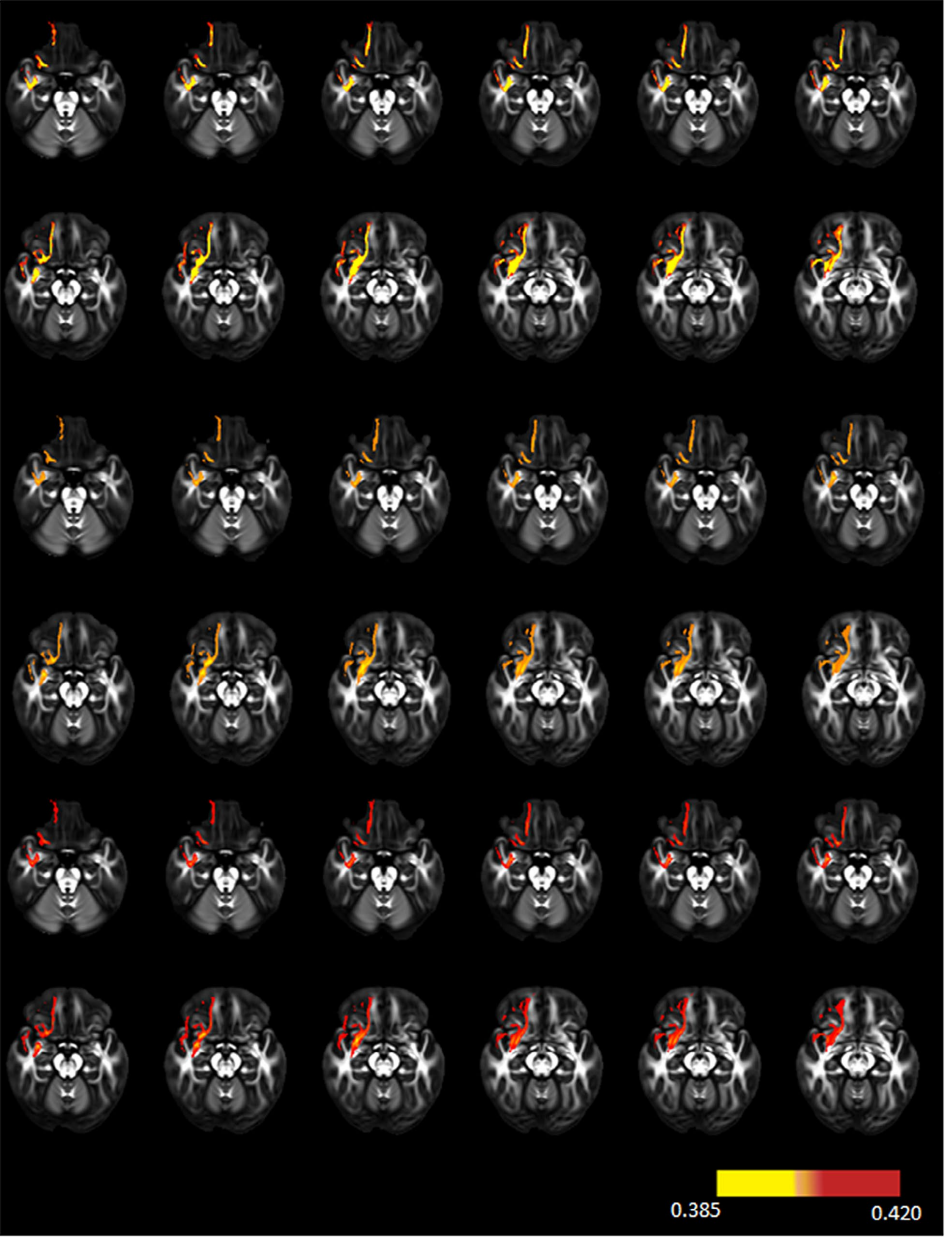
It shows the reconstruction of the right uncinate fascicles of 3 exemplarily selected participants with different FA values in diffusion tensor imaging. Yellow values indicate low FA, while red values indicate high FA

The results of the DTI data from patients with prolactinoma and healthy controls are shown in Supplementary Table 1. Of the 42 tracts analyzed between the two groups with ANOVA, there was a significant difference only in the right uncinate fasciculus (R-UF) tract (*p* = 0.035). Lower FA values were found in patients with prolactinoma compared to healthy controls, indicating axonal damage (0.407 ± 0.021 vs. 0.417 ± 0.019). After applying the Bonferroni correction, the two groups had no significant difference in 42 tracts (*p* > 0.0012 for all). For example, Fig. 2 shows the reconstruction of the R-UF of 3 participants with different FA values in DTI.

### Relationship between neuropsychological tests and clinical-sociodemographic findings

In patients with prolactinoma, no correlation was found between the neuropsychological test results and age and sex (*p* > 0.05 for both). For all parameters of cognitive performance, an increase was seen with increasing education (*p* < 0.05 for all). There were no differences in cognitive function between patients treated with dopamine agonists at the time of the study and patients who had discontinued this treatment for at least two months (Supplementary Table 2). There was no correlation between the initial prolactin level, adenoma size, current prolactin level, cumulative cabergoline dose, and the results of the neuropsychological tests (*p* > 0.05 for all). However, when patients were grouped according to their prolactin level, the score for short-term memory was 0.98 ± 0.01 in patients with normoprolactinemia, 0.75 ± 0.25 in patients with hyperprolactinemia and 0.63 ± 0.51 in patients with hypoprolactinemia (*p* = 0.005).

### Relationship between DTI findings and clinical-sociodemographic findings

The R-UF tract in which significant differences with ANOVA test were found between patients with prolactinoma and healthy controls in the DTI examination was independent of initial prolactin level, adenoma size, current prolactin level, cumulative cabergoline dose, age, sex, and education (*p* > 0.05, for all). There were no differences in DTI results between patients treated with dopamine agonists at the time of the study and patients who had discontinued this treatment for at least two months (Supplementary Table 3). There were significant differences in comparing R-UF FA values between the patient groups depending on the prolactin level. The R-UF FA values were found to be 0.387 ± 0.023 in patients with hypoprolactinemia, 0.411 ± 0.027 in patients with normoprolactinemia, and 0.397 ± 0.021 in patients with hyperprolactinemia (*p* = 0.001).

### Relationship between DTI findings and neuropsychological tests

The FA values of the R-UF tract determined in patients with prolactinoma in the DTI examination and the scores of the neuropsychological tests determined in patients with prolactinoma were subjected to a correlation analysis. The significant correlations found are listed in Table 4. As the FA values in the R-UF tract decreased, the patients’ scores for long-term memory, the category test, and the letter fluency test decreased, while their anxiety level increased.

**Table 4:** The correlation analysis between patients’ neuropsychiatric tests and right uncinate fasciculus tract FA values measured in DTI

Neuropsychological tests	Right uncinate fasciculus FA values	
	*r*-values	*p*-values
Long-Term Memory	0,246	0,036
Category Test	0,363	0,002
Letter Fluency Test	0,287	0,014
Beck Anxiety Scale	-0,236	0,045

DTI, diffusion tensor imaging; FA, fractioned anisotropy; r-value, correlation coefficient

## Discussion

In this study, a comprehensive assessment of cognitive function, clinical and sociodemographic characteristics, and detailed radiologic imaging of patients with prolactinoma was performed. Patients with prolactinoma were found to have impaired cognitive function compared to the healthy population. Long-term, visual and verbal memory, attention, concentration, and language functions were lower than in the healthy group. While patients with normoprolactinemia had higher short-term memory scores, hypoprolactinemia, and hyperprolactinemia had the lowest scores. When evaluated with DTI, the R-UF tract was impaired in patients with prolactinoma compared to healthy controls, but no significant difference between the tracts was found after multiple corrections. The low FA values in the R-UF tract were associated with poorer scores on neuropsychological tests. In addition, measured R-UF FA values were lowest in patients with hypoprolactinemia and highest in patients with normoprolactinemia.

The observation that cognitive abilities are impaired in patients with prolactinoma has attracted the attention of clinicians and has become the subject of research. Bala et al. reported that the attention and working memory of patients with prolactinoma are lower than in the normal population. They explained that the impairment of cognitive function is related to the prolactin level but not to the size of the adenoma [24]. Another study reported that patients with prolactinoma performed worse on tests of verbal and nonverbal memory and attention than healthy controls, emphasizing the negative effects of high prolactin levels on cognitive function [4]. Chen et al. reported that patients with prolactinoma showed slower responses and impaired attentional processing abilities. This study emphasizes the inverse relationship between cognitive function and prolactin levels [25]. Montalvo et al. prospectively followed seven patients with prolactinoma for cognitive function. They subjected the patients to a cognitive assessment at the time of diagnosis and one year after starting treatment with cabergoline. They showed significant improvements after treatment compared to baseline in processing speed, working memory, visual learning, reasoning, and problem-solving ability. They cited the normalization of prolactin levels and possible procognitive effects of cabergoline as potential mechanisms for this improvement [26]. In this study, we subjected patients with prolactinoma to a comprehensive assessment of cognitive function. The result was that the long-term visual and verbal memory, attention, concentration, and language functions of patients with prolactinoma were impaired compared to the healthy population. These results emphasize the presence of cognitive dysfunction in patients with prolactinoma reported in the literature. We also found that short-term memory was impaired in patients with hyper- and hypoprolactinemia, especially in patients with hypoprolactinemia. It is well known that low prolactin levels are associated with decreased sexual desire, sexual dysfunction, and reduced quality of life [27–29]. However, this study is significant in showing that prolactin levels that are too low and too high negatively affect cognitive function for the first time. This finding emphasizes that clinicians should avoid overtreatment of patients with prolactinoma.

The mechanism underlying the cognitive impairment observed in patients with prolactinoma is still unclear. Many studies reported an association between elevated prolactin and cognitive impairment but did not specify how it affects cognitive function [24–26]. Cao et al. investigated the cause of cognitive impairment in patients with prolactinoma using attention EEG. They reported that patients with prolactinoma showed greater frontoparietal theta and alpha coherence in the right lateral hemisphere and that this increase in coherence correlated with prolactin levels. It has been suggested that increased frontoparietal alpha and theta coherence is a marker of poor attentional processing in patients with prolactinoma [30]. Another study found that response activation and inhibition measured by EEG were lower in patients with prolactinoma than in healthy controls. They found that this was associated with decreased frontal theta wave oscillation and that prolactin elevation and theta wave oscillation were inversely related. They concluded that this may be the underlying mechanism of cognitive dysfunction in patients with prolactinoma [6]. In one study, patients with prolactinoma were reported to have impaired cognitive flexibility and lower performance in task situations. EEG showed decreased frontal theta energy as a marker of this cognitive dysfunction. It was also reported that frontoparietal connectivity was impaired [5]. Yao et al. found that cognitive impairment in female patients with prolactinoma was associated with a decrease in brain gray matter volume. A correlation was found between decreased brain volume and prolactin level [7]. Since the volume of brain gray matter has been reported to be reduced in patients with prolactinoma and connectivity between the centers of cognitive functions is impaired, the need for structural assessment of the pathways in patients with prolactinoma has become apparent.

The main pathways connecting the cortex regions in the brain where cognitive functions are carried out are the pathways in the brain’s white matter [31]. DTI is an imaging technique that can assess white matter microstructures in the central nervous system. DTI measures the directionality of water molecule diffusion to generate tissue contrasts that can be used to evaluate axonal organization in the central nervous system [8]. The directional information can be used to select and trace ascending and descending pathways in the brain, known as tractography [23]. This study examined white matter tracts in the brains of patients with prolactinoma and healthy controls for the first time and compared them with DTI. We found lower FA values in the R-UF tract in patients with prolactinoma compared to the healthy population, indicating neuronal damage. However, no significant difference was found between the tracts after multiple corrections for the measured low FA values. The UF is a bidirectional, long-range white matter pathway that connects the lateral orbitofrontal cortex and Brodmann area 10 to the anterior temporal lobe [32]. Abnormalities in the UF are associated with various psychiatric disorders and play a role in memory, language, and social-emotional processing [33]. In our study, neuropsychological tests showed that long-term visual and verbal memory, attention, concentration, and language functions were impaired in patients with prolactinoma compared to the healthy population. In addition, a correlation was found between the FA values measured in the R-UF tract and the results of the neuropsychological tests. Another finding was that the lowest FA levels of R-UF measurements occurred in patients with hypoprolactinemia and the highest in patients with normoprolactinemia. These results suggest that high and too-low levels of prolactin may lead to cognitive dysfunction by causing structural disruption of brain tracts.

An explanation is why a difference was only found in R-UF among the 42 tracts evaluated. One possible explanation is that UF is one of the last tracts of white matter to reach its peak of maturation, with the developmental period completed at 28–35 years of age [34]. In our study population, the age of patients was 30 ± 7 years, and the UF tract may have been more affected by prolactinoma than others due to its progressive development. However, the reason for the observed right-left lateralization is unclear. Similarly, different lateralization findings are reported in the small but growing literature on UF [35–37]. One possible explanation could be that we recruited right-hand dominant participants to ensure the most significant possible standardization of the study. All results and explanations are hypothetical, and further studies are needed.

The prolactin mediates neuroprotection, plays a role in the proliferation of oligodendrocyte precursor cells, releases neurotrophic factors, and increases white matter volume [38–46]. In their study, Paul et al. reported that moderately high prolactin levels reduced retinal nerve fiber layer damage in patients with pituitary macroadenoma and compression of the optic chiasm. In contrast, the opposite effect was observed at very high and low prolactin levels [43]. In addition, the protective role of prolactin in white matter damage has been demonstrated in patients with multiple sclerosis and mouse models of spinal cord injury [44–46]. These studies link increased oligodendrocyte proliferation and remyelination after injury to increased prolactin levels and white matter volume [44–46]. In another study, remyelination after anterior visual pathway compression injury in a patient with empty sella syndrome was shown to be sensitive to changes in serum prolactin levels [47]. The low FA values observed in our study of the neurons that form the tracts in the white matter of the brain responsible for cognitive function in patients with prolactinoma underscores the relationship between high prolactin and neurotoxicity or neuroprotection previously reported in other studies. On the other hand, we report that lower than normal prolactin levels may also cause damage to neurons in the white matter of the brain and corresponding cognitive dysfunction, which has not been previously reported.

This study has some limitations. First, the study’s cross-sectional nature and the fact that the patients participated at different stages, such as using cabergoline or not, during their treatment. However, we applied strict exclusion criteria to rule out many conditions that could affect the assessment of cognitive function. This allowed us to ensure that the results obtained could be attributed to prolactinoma. Furthermore, by recruiting patients at different treatment stages, we assessed the impact of treatment outcomes on cognitive function. Another limitation was that the findings could indicate neuronal damage and could not be confirmed at the pathological and molecular levels. However, the detailed clinical, radiological, and neuropsychological evaluation provided new insights and a different perspective for understanding cognitive dysfunction in patients with prolactinoma.

## Conclusion

This study confirmed the presence of cognitive impairment in patients with prolactinoma, with scores for long-term, visual, and verbal memory, attention, concentration, and language function being lower than in healthy controls. To understand the cause of cognitive dysfunction, the microarchitecture of the neural pathways in the brain was investigated using DTI. This is the first time that cognitive dysfunction in patients with prolactinoma has been studied using this method. According to the DTI results, no significant structural changes were found in patients with prolactinoma brain tracts. Still, there may be a link between potential damage in the R-UF and cognitive dysfunction, and further research is needed. It was also found that the highest cognitive functions in patients with prolactinoma were achieved with normoprolactinemia, while the development of hypoprolactinemia was associated with the lowest cognitive functions. Patients with prolactinoma also emphasized that overtreatment should be avoided.

## Electronic supplementary material

Below is the link to the electronic supplementary material.

Supplementary Material 1

## Data Availability

All data obtained or analyzed in this study are included in this article [and] its tables. The data archive can be made available on request. Further requests can be directed to the corresponding author.

## References

[1] Petersenn S, Fleseriu M, Casanueva FF et al (2023) Diagnosis and management of prolactin-secreting pituitary adenomas: a Pituitary Society international Consensus Statement [published correction appears in nat Rev Endocrinol. 2023]. Nat Rev Endocrinol 19(12):722–740. 10.1038/s41574-023-00886-5 37670148 10.1038/s41574-023-00886-5

[2] Auriemma RS, Pirchio R, Pivonello C, Garifalos F, Colao A, Pivonello R (2023) Approach to the patient with Prolactinoma. J Clin Endocrinol Metab 108(9):2400–2423. 10.1210/clinem/dgad174 36974474 10.1210/clinem/dgad174 PMC10438891

[3] Lopez-Vicchi F, De Winne C, Brie B, Sorianello E, Ladyman SR, Becu-Villalobos D (2020) Metabolic functions of prolactin: physiological and pathological aspects. J Neuroendocrinol 32(11):e12888. 10.1111/jne.12888 33463813 10.1111/jne.12888

[4] Bala A, Łojek E, Marchel A (2016) Cognitive functioning of patients with a PRL-secreting pituitary adenoma: a preliminary report. Neurology 86(8):731–734. 10.1212/WNL.0000000000002252 26701376 10.1212/WNL.0000000000002252

[5] Cao C, Wen W, Chen A et al (2023) Neuropsychological alterations of Prolactinomas’ cognitive flexibility in Task switching. Brain Sci 13(1):82. 10.3390/brainsci13010082. Published 2023 Jan 1 36672063 10.3390/brainsci13010082 PMC9856801

[6] Cao C, Wen W, Liu B et al (2020) Theta oscillations in prolactinomas: neurocognitive deficits in executive controls. Neuroimage Clin 28:102455. 10.1016/j.nicl.2020.102455 33038668 10.1016/j.nicl.2020.102455 PMC7554198

[7] Yao S, Song J, Gao J et al (2018) Cognitive Function and Serum Hormone Levels Are Associated with Gray Matter Volume Decline in Female Patients with Prolactinomas. *Front Neurol*. ;8:742. Published 2018 Jan 29. 10.3389/fneur.2017.00742 10.3389/fneur.2017.00742 PMC5797301 29434564

[8] Assaf Y, Pasternak O (2008) Diffusion tensor imaging (DTI)-based white matter mapping in brain research: a review. J Mol Neurosci 34(1):51–61. 10.1007/s12031-007-0029-0 18157658 10.1007/s12031-007-0029-0

[9] Melmed S, Casanueva FF, Hoffman AR et al (2011) Diagnosis and treatment of hyperprolactinemia: an endocrine Society clinical practice guideline. J Clin Endocrinol Metab 96(2):273–288. 10.1210/jc.2010-1692 21296991 10.1210/jc.2010-1692

[10] Selekler K, Cangöz B, Uluç S (2010) Power of discrimination of Montreal Cognitive Assessment (MOCA) scale in Turkish patients with mild cognitive impairment and Alzheimer’s disease. Turkish J Geriatr 13(3):166–171

[11] Scarpina F, Tagini S (2017) The Stroop Color and Word Test. Front Psychol 8:557 Published 2017 Apr 12. 10.3389/fpsyg.2017.00557 28446889 10.3389/fpsyg.2017.00557 PMC5388755

[12] Savaş DDE, Yerlikaya D, Yener GG, Tanör ÖÖ (2020) Validity, reliability and normative data of the Stroop Test Įapa Version. Turk Psikiyatri Derg 31(1):9–21 32594475 10.5080/u23549

[13] Öktem Ö (1992) Sözel bellek süreçleri testi, bir ön çalışma. Nöropsikiyatri Arşivi 29(4):196–206

[14] Henderson SK, Peterson KA, Patterson K, Lambon Ralph MA, Rowe JB (2023) Verbal fluency tests assess global cognitive status but have limited diagnostic differentiation: evidence from a large-scale examination of six neurodegenerative diseases. Brain Commun 5(2):fcad042 Published 2023 Feb 21. 10.1093/braincomms/fcad042 36910418 10.1093/braincomms/fcad042 PMC9999359

[15] Karakaş S, Kafadar H, Eski R (1996) Wechsler bellek ölçeği geliştirilmiş formunun testtekrar test güvenirliği. Türk Psikoloji Dergisi 11(38):46–52

[16] Julian LJ (2011) Measures of anxiety: state-trait anxiety inventory (STAI), Beck anxiety inventory (BAI), and hospital anxiety and depression scale-anxiety (HADS-A). Arthritis Care Res (Hoboken) 63(Suppl 11):S467–S472. 10.1002/acr.20561 22588767 10.1002/acr.20561 PMC3879951

[17] Ulusoy M, Sahin NH, Erkmen H (1998) Turkish version of the Beck anxiety inventory: psychometric properties. J Cogn Psychother 12(2):163

[18] Smarr KL, Keefer AL (2011) Measures of depression and depressive symptoms: Beck Depression Inventory-II (BDI-II), Center for epidemiologic studies Depression Scale (CES-D), geriatric Depression Scale (GDS), hospital anxiety and Depression Scale (HADS), and Patient Health Questionnaire-9 (PHQ-9). Arthritis Care Res (Hoboken) 63(Suppl 11):S454–S466. 10.1002/acr.20556 22588766 10.1002/acr.20556

[19] Andersson JL, Skare S, Ashburner J (2003) How to correct susceptibility distortions in spin-echo echo-planar images: application to diffusion tensor imaging. NeuroImage 20(2):870–888. 10.1016/S1053-8119(03)00336-7 14568458 10.1016/S1053-8119(03)00336-7

[20] Andersson JLR, Sotiropoulos SN (2016) An integrated approach to correction for off-resonance effects and subject movement in diffusion MR imaging. NeuroImage 125:1063–1078. 10.1016/j.neuroimage.2015.10.019 26481672 10.1016/j.neuroimage.2015.10.019 PMC4692656

[21] Smith SM (2002) Fast robust automated brain extraction. Hum Brain Mapp 17(3):143–155. 10.1002/hbm.10062 12391568 10.1002/hbm.10062 PMC6871816

[22] Veraart J, Sijbers J, Sunaert S, Leemans A, Jeurissen B (2013) Weighted linear least squares estimation of diffusion MRI parameters: strengths, limitations, and pitfalls. NeuroImage 81:335–346. 10.1016/j.neuroimage.2013.05.028 23684865 10.1016/j.neuroimage.2013.05.028

[23] Warrington S, Bryant KL, Khrapitchev AA et al (2020) XTRACT - standardised protocols for automated tractography in the human and macaque brain. NeuroImage 217:116923. 10.1016/j.neuroimage.2020.116923 32407993 10.1016/j.neuroimage.2020.116923 PMC7260058

[24] Bala A, Dziedzic T, Olejnik A, Marchel A (2022) Attention and working memory in patients with prolactinomas: a case-control study. Sci Rep 12(1):22565 Published 2022 Dec 29. 10.1038/s41598-022-26331-7 36581642 10.1038/s41598-022-26331-7 PMC9800401

[25] Chen A, Cao C, Liu B et al (2022) Hyperprolactinemia Associated with Attentional Processing and Interference Control Impairments in patients with Prolactinomas. Brain Sci 12(8):1091. 10.3390/brainsci12081091. Published 2022 Aug 17 36009154 10.3390/brainsci12081091 PMC9406026

[26] Montalvo I, Llorens M, Caparrós L et al (2018) Improvement in cognitive abilities following cabergoline treatment in patients with a prolactin-secreting pituitary adenoma. Int Clin Psychopharmacol 33(2):98–102. 10.1097/YIC.0000000000000199 29035904 10.1097/YIC.0000000000000199

[27] Corona G, Wu FC, Rastrelli G et al (2014) Low prolactin is associated with sexual dysfunction and psychological or metabolic disturbances in middle-aged and elderly men: the European Male Aging Study (EMAS). J Sex Med 11(1):240–253. 10.1111/jsm.12327 24345293 10.1111/jsm.12327

[28] Peixoto C, Carrilho CG, Ribeiro TTSB et al (2019) Relationship between sexual hormones, quality of life and postmenopausal sexual function. Trends Psychiatry Psychother 41(2):136–143 Published 2019 May 30. 10.1590/2237-6089-2018-0057 31166564 10.1590/2237-6089-2018-0057

[29] Bohnet HG, Mühlenstedt D, Hanker JP, Schneider HP (1977) Prolactin oversuppression. Arch Gynakol 223(3):173–178. 10.1007/BF00667386 579295 10.1007/BF00667386

[30] Cao C, Wang Y, Liu J et al (2021) Altered connectivity of the Frontoparietal Network during attention Processing in Prolactinomas. Front Neurol 12:638851 Published 2021 Aug 30. 10.3389/fneur.2021.638851 34526949 10.3389/fneur.2021.638851 PMC8435841

[31] Schmahmann JD, Smith EE, Eichler FS, Filley CM (2008) Cerebral white matter: neuroanatomy, clinical neurology, and neurobehavioral correlates. Ann N Y Acad Sci 1142:266–309. 10.1196/annals.1444.017 18990132 10.1196/annals.1444.017 PMC3753195

[32] Von Der Heide RJ, Skipper LM, Klobusicky E, Olson IR (2013) Dissecting the uncinate fasciculus: disorders, controversies and a hypothesis. Brain 136(Pt 6):1692–1707. 10.1093/brain/awt094 23649697 10.1093/brain/awt094 PMC3673595

[33] Coad BM, Postans M, Hodgetts CJ, Muhlert N, Graham KS, Lawrence AD (2020) Structural connections support emotional connections: Uncinate Fasciculus microstructure is related to the ability to decode facial emotion expressions. Neuropsychologia 145:106562. 10.1016/j.neuropsychologia.2017.11.006 29122609 10.1016/j.neuropsychologia.2017.11.006 PMC7534036

[34] Olson IR, Von Der Heide RJ, Alm KH, Vyas G (2015) Development of the uncinate fasciculus: implications for theory and developmental disorders. Dev Cogn Neurosci 14:50–61. 10.1016/j.dcn.2015.06.003 26143154 10.1016/j.dcn.2015.06.003 PMC4795006

[35] Hasan KM, Iftikhar A, Kamali A et al (2009) Development and aging of the healthy human brain uncinate fasciculus across the lifespan using diffusion tensor tractography. Brain Res 1276:67–76. 10.1016/j.brainres.2009.04.025 19393229 10.1016/j.brainres.2009.04.025 PMC2693464

[36] Kitamura S, Morikawa M, Kiuchi K et al (2011) Asymmetry, sex differences and age-related changes in the white matter in the healthy elderly: a tract-based study. BMC Res Notes 4:378. 10.1186/1756-0500-4-378. Published 2011 Oct 4 21970546 10.1186/1756-0500-4-378 PMC3205060

[37] Kubicki M, Westin CF, Maier SE et al (2002) Uncinate fasciculus findings in schizophrenia: a magnetic resonance diffusion tensor imaging study. Am J Psychiatry 159(5):813–820. 10.1176/appi.ajp.159.5.813 11986136 10.1176/appi.ajp.159.5.813 PMC2803760

[38] Cabrera-Reyes EA, Limón-Morales O, Rivero-Segura NA, Camacho-Arroyo I, Cerbón M (2017) Prolactin function and putative expression in the brain. Endocrine 57(2):199–213. 10.1007/s12020-017-1346-x 28634745 10.1007/s12020-017-1346-x

[39] Tani N, Ikeda T, Ishikawa T (2024) Effects of Prolactin on Brain neurons under Hypoxia. Life (Basel) 14(1):152. 10.3390/life14010152. Published 2024 Jan 21 38276281 10.3390/life14010152 PMC10817236

[40] Knudtzon J, Bogsnes A, Norman N (1989) Changes in prolactin and growth hormone levels during hypoxia and exercise. Horm Metab Res 21(8):453–454. 10.1055/s-2007-1009260 2793067 10.1055/s-2007-1009260

[41] Zhang YS, Du JZ (2000) The response of growth hormone and prolactin of rats to hypoxia. Neurosci Lett 279(3):137–140. 10.1016/s0304-3940(99)00968-4 10688048 10.1016/s0304-3940(99)00968-4

[42] Richalet JP, Letournel M, Souberbielle JC (2010) Effects of high-altitude hypoxia on the hormonal response to hypothalamic factors. Am J Physiol Regul Integr Comp Physiol 299(6):R1685–R1692. 10.1152/ajpregu.00484.2010 20926759 10.1152/ajpregu.00484.2010

[43] Paul DA, Rodrigue A, Contento N et al (2022) Prolactin at moderately increased levels confers a neuroprotective effect in non-secreting pituitary macroadenomas. PLoS ONE 17(8):e0271690 Published 2022 Aug 3. 10.1371/journal.pone.0271690 35921360 10.1371/journal.pone.0271690 PMC9348739

[44] Gregg C, Shikar V, Larsen P et al (2007) White matter plasticity and enhanced remyelination in the maternal CNS. J Neurosci 27(8):1812–1823. 10.1523/JNEUROSCI.4441-06.2007 17314279 10.1523/JNEUROSCI.4441-06.2007 PMC6673564

[45] Anagnostou I, Reyes-Mendoza J, Morales T (2018) Glial cells as mediators of protective actions of prolactin (PRL) in the CNS. Gen Comp Endocrinol 265:106–110. 10.1016/j.ygcen.2018.01.024 29378204 10.1016/j.ygcen.2018.01.024

[46] De Giglio L, Marinelli F, Prosperini L et al (2015) Relationship between Prolactin plasma levels and White Matter volume in women with multiple sclerosis. Mediators Inflamm 2015:732539. 10.1155/2015/732539 26236110 10.1155/2015/732539 PMC4510259

[47] Paul DA, Strawderman E, Rodriguez A et al (2021) Empty Sella Syndrome as a window into the neuroprotective effects of Prolactin. Front Med (Lausanne) 8:680602 Published 2021 Jul 8. 10.3389/fmed.2021.680602 34307410 10.3389/fmed.2021.680602 PMC8295462

