# Supplementary material for: Investigating the relationship between cognitive impairment and brain white matter tracts using diffusion tensor imaging in patients with prolactinoma
Source: J Endocrinol Invest. 2024 Oct 3;48(2):345–55. doi: 10.1007/s40618-024-02442-y (PMC11785683; doi:10.1007/s40618-024-02442-y)
Supplement: Supplementary file 1 — Supplementary Material 1 [file 40618_2024_2442_MOESM1_ESM.docx]

| **Supplementary Table 1.** The comparison of data obtained as a result of diffusion tensor imaging FA values in participants’ | | | |
| --- | --- | --- | --- |
| **White matter tracts, mean ± SD** | **Patients with Prolactinoma (n=37)** | **Healthy Controls (n=37)** | **p-values*** |
| Anterior commissure | 0.269 ± 0.046 | 0.275 ± 0.045 | 0.615 |
| Left arcuate fasciculus | 0.471 ± 0.018 | 0.477 ± 0.026 | 0.280 |
| Right arcuate fasciculus | 0.451 ± 0.014 | 0.451 ± 0.023 | 0.686 |
| Left acoustic radiation | 0.401 ± 0.021 | 0.407 ± 0.021 | 0.132 |
| Right acoustic radiation | 0.389 ± 0.021 | 0.389 ± 0.025 | 0.989 |
| Left anterior thalamic radiation | 0.428 ± 0.014 | 0.424 ± 0.022 | 0.375 |
| Right anterior thalamic radiation | 0.412 ± 0.015 | 0.414 ± 0.021 | 0.573 |
| Left cingulum subsection: dorsal | 0.476 ± 0.028 | 0.476 ± 0.023 | 0.994 |
| Right cingulum subsection: dorsal | 0.431 ± 0.024 | 0.427 ± 0.033 | 0.799 |
| Left cingulum subsection: peri-genual | 0.401 ± 0.028 | 0.398 ± 0.034 | 0.792 |
| Right cingulum subsection: peri-genual | 0.362 ± 0.031 | 0.368 ± 0.042 | 0.356 |
| Left cingulum subsection temporal | 0.338 ± 0.038 | 0.347 ± 0.026 | 0.223 |
| Right cingulum subsection temporal | 0.332 ± 0.032 | 0.337 ± 0.035 | 0.522 |
| Left corticospinal tract | 0.555 ± 0.024 | 0.545 ± 0.027 | 0.148 |
| Right corticospinal tract | 0.561 ± 0.021 | 0.553 ± 0.023 | 0.134 |
| Left frontal aslant | 0.426 ± 0.018 | 0.426 ± 0.026 | 0.904 |
| Right frontal aslant | 0.422 ± 0.021 | 0.417 ± 0.025 | 0.421 |
| Forceps major | 0.591 ± 0.026 | 0.591 ± 0.026 | 0.549 |
| Forceps minor | 0.529 ± 0.027 | 0.536 ± 0.031 | 0.262 |
| Left fornix | 0.313 ± 0.028 | 0.309 ± 0.031 | 0.546 |
| Right fornix | 0.312 ± 0.038 | 0.323 ± 0.042 | 0.314 |
| Left inferior fronto-occipital fasciculus | 0.499 ± 0.017 | 0.497 ± 0.021 | 0.805 |
| Right inferior fronto-occipital fasciculus | 0.488 ± 0.016 | 0.489 ± 0.021 | 0.665 |
| Left inferior longitudinal fasciculus | 0.435 ± 0.021 | 0.439 ± 0.028 | 0.483 |
| Right inferior longitudinal fasciculus | 0.444 ± 0.026 | 0.446 ± 0.023 | 0.769 |
| Middle cerebellar peduncle | 0.531 ± 0.034 | 0.540 ± 0.031 | 0.222 |
| Left middle longitudinal fasciculus | 0.456 ± 0.021 | 0.457 ± 0.024 | 0.916 |
| Right middle longitudinal fasciculus | 0.451 ± 0.019 | 0.447 ± 0.023 | 0.541 |
| Left optic radiation | 0.524 ± 0.018 | 0.524 ± 0.024 | 0.914 |
| Right optic radiation | 0.505 ± 0.021 | 0.505 ± 0.023 | 0.815 |
| Left superior longitudinal fasciculus 1 | 0.441 ± 0.039 | 0.437 ± 0.031 | 0.813 |
| Right superior longitudinal fasciculus 1 | 0.433 ± 0.029 | 0.434 ± 0.033 | 0.811 |
| Left superior longitudinal fasciculus 2 | 0.378 ± 0.026 | 0.383 ± 0.025 | 0.487 |
| Right superior longitudinal fasciculus 2 | 0.386 ± 0.026 | 0.389 ± 0.025 | 0.548 |
| Left superior longitudinal fasciculus 3 | 0.419 ± 0.025 | 0.426 ± 0.026 | 0.154 |
| Right superior longitudinal fasciculus 3 | 0.428 ± 0.023 | 0.428 ± 0.021 | 0.995 |
| Left superior thalamic radiation | 0.462 ± 0.018 | 0.461 ± 0.026 | 0.859 |
| Right superior thalamic radiation | 0.442 ± 0.019 | 0.441 ± 0.022 | 1.000 |
| Left uncinate fasciculus | 0.416 ± 0.022 | 0.421 ± 0.021 | 0.275 |
| Right uncinate fasciculus | 0.407 ± 0.021 | 0.417 ± 0.019 | 0.035 |
| Left vertical occipital fasciculus | 0.408 ± 0.029 | 0.411 ± 0.025 | 0.722 |
| Right vertical occipital fasciculus | 0.403 ± 0.027 | 0.403 ± 0.028 | 0.827 |

FA, Fractional anisotropy; SD, standard deviation

* The p values were obtained using a one-way analysis of covariance (ANCOVA) with the adjustment for age and sex covariance. Significance level when Bonferroni correction is applied: p < 0.05/42 = 0.0012.

**Supplementary Table 2.** Comparison of scores obtained from Verbal Memory Processes Test, Category and Letter Fluency tests, Stroop test, MoCA test, and WMS tests according to dopamine agonist use in patients with prolactinoma

| **Tests, mean ± SD** | **DA use at the time of the examination or within two months before** | | **p-values*** |
| --- | --- | --- | --- |
|  | **No (n=10)** | **Yes (n=27)** |  |
| Verbal Memory Processes Test | | | |
| Short-Term Memory Score | 6.0 ± 1.2 | 6.6 ± 1.7 | 0.377 |
| Learning Score | 120.3 ± 13.9 | 122.2 ± 14.1 | 0.742 |
| High Learning Score | 14.4 ± 0.9 | 14.6 ± 0.8 | 0.631 |
| Long-Term Memory Score | 13.0 ± 1.3 | 12.5 ± 1.6 | 0.487 |
| Total Memory Score | 15.0 ± 0.1 | 14.9 ± 0.3 | 0.320 |
| Category Test Score | 20.7 ± 2.4 | 20.6 ± 4.4 | 0.948 |

| Letter Fluency Test Score | 29.1 ± 8.0 | 28.8 ± 10.1 | 0.941 |
| --- | --- | --- | --- |
| Stroop Test Score | 42.8 ± 11.7 | 40.5 ± 10.5 | 0.610 |
| MoCA Test Score | 25.4 ± 3.1 | 23.9 ± 3.3 | 0.258 |
| WMS Total Score | 13.0 ± 1.2 | 11.9 ± 1.9 | 0.132 |

MoCA, Montreal Cognitive Assessment Test; SD, standard deviation; WMS, Wechsler Memory Scale Visual Production Subtest

* The p values were obtained using a one-way analysis of covariance (ANCOVA) with the adjustment for age and sex covariance. Significance level when Bonferroni correction is applied: p < 0.05/10 = 0.005.

Category Test, the number of animals counted in 1 minute; Letter Fluency Test, the total number of words starting with the letters K, A, and S counted in one minute; Stroop Test; The difference in time between reading the color of the word and reading the word itself

| **Supplementary Table 3.** Comparison of data obtained as a result of diffusion tensor imaging FA values ​​of patients with prolactinoma according to dopamine agonist use | | | |
| --- | --- | --- | --- |
| **White matter tracts, mean ± SD** | **DA use at the time of the examination or within two months before** | | **p-values*** |
|  | **No (n=10)** | **Yes (n=27)** |  |
| Anterior commissure | 0.263 ± 0.041 | 0.271 ± 0.048 | 0.666 |
| Left arcuate fasciculus | 0.471 ± 0.027 | 0.471 ± 0.017 | 0.998 |
| Right arcuate fasciculus | 0.438 ± 0.014 | 0.442 ± 0.013 | 0.616 |
| Left acoustic radiation | 0.397 ± 0.021 | 0.399 ± 0.020 | 0.816 |
| Right acoustic radiation | 0.390 ± 0.018 | 0.388 ± 0.022 | 0.912 |
| Left anterior thalamic radiation | 0.427 ± 0.009 | 0.428 ± 0.016 | 0.842 |
| Right anterior thalamic radiation | 0.408 ± 0.010 | 0.413 ± 0.017 | 0.547 |
| Left cingulum subsection: dorsal | 0.483 ± 0.022 | 0.474 ± 0.029 | 0.468 |
| Right cingulum subsection: dorsal | 0.440 ± 0.021 | 0.427 ± 0.023 | 0.202 |
| Left cingulum subsection: peri-genual | 0.401 ± 0.028 | 0.400 ± 0.030 | 0.950 |
| Right cingulum subsection: peri-genual | 0.349 ± 0.014 | 0.363 ± 0.032 | 0.287 |
| Left cingulum subsection temporal | 0.348 ± 0.027 | 0.335 ± 0.041 | 0.442 |
| Right cingulum subsection temporal | 0.349 ± 0.021 | 0.328 ± 0.034 | 0.134 |
| Left corticospinal tract | 0.561 ± 0.014 | 0.552 ± 0.025 | 0.415 |
| Right corticospinal tract | 0.562 ± 0.025 | 0.560 ± 0.021 | 0.885 |
| Left frontal aslant | 0.433 ± 0.021 | 0.423 ± 0.017 | 0.217 |
| Right frontal aslant | 0.419 ± 0.009 | 0.422 ± 0.022 | 0.704 |
| Forceps major | 0.597 ± 0.018 | 0.588 ± 0.027 | 0.457 |
| Forceps minor | 0.524 ± 0.022 | 0.529 ± 0.028 | 0.641 |
| Left fornix | 0.301 ± 0.016 | 0.316 ± 0.031 | 0.186 |
| Right fornix | 0.298 ± 0.033 | 0.317 ± 0.039 | 0.246 |
| Left inferior fronto-occipital fasciculus | 0.494 ± 0.021 | 0.501 ± 0.016 | 0.460 |
| Right inferior fronto-occipital fasciculus | 0.482 ± 0.017 | 0.489 ± 0.016 | 0.317 |
| Left inferior longitudinal fasciculus | 0.438 ± 0.017 | 0.434 ± 0.021 | 0.657 |
| Right inferior longitudinal fasciculus | 0.437 ± 0.027 | 0.446 ± 0.027 | 0.429 |
| Middle cerebellar peduncle | 0.534 ± 0.043 | 0.529 ± 0.033 | 0.748 |
| Left middle longitudinal fasciculus | 0.454 ± 0.022 | 0.456 ± 0.021 | 0.830 |
| Right middle longitudinal fasciculus | 0.450 ± 0.023 | 0.451 ± 0.018 | 0.942 |
| Left optic radiation | 0.517 ± 0.031 | 0.525 ± 0.014 | 0.305 |
| Right optic radiation | 0.497 ± 0.030 | 0.506 ± 0.017 | 0.301 |
| Left superior longitudinal fasciculus 1 | 0.449 ± 0.029 | 0.436 ± 0.041 | 0.456 |
| Right superior longitudinal fasciculus 1 | 0.438 ± 0.018 | 0.431 ± 0.031 | 0.581 |
| Left superior longitudinal fasciculus 2 | 0.389 ± 0.024 | 0.376 ± 0.026 | 0.268 |
| Right superior longitudinal fasciculus 2 | 0.390 ± 0.023 | 0.385 ± 0.027 | 0.644 |
| Left superior longitudinal fasciculus 3 | 0.426 ± 0.018 | 0.416 ± 0.025 | 0.309 |
| Right superior longitudinal fasciculus 3 | 0.419 ± 0.022 | 0.430 ± 0.023 | 0.290 |
| Left superior thalamic radiation | 0.468 ± 0.009 | 0.459 ± 0.019 | 0.274 |
| Right superior thalamic radiation | 0.453 ± 0.017 | 0.438 ± 0.018 | 0.060 |
| Left uncinate fasciculus | 0.417 ± 0.026 | 0.415 ± 0.022 | 0.872 |
| Right uncinate fasciculus | 0.407 ± 0.014 | 0.406 ± 0.021 | 0.937 |
| Left vertical occipital fasciculus | 0.413 ± 0.036 | 0.407 ± 0.028 | 0.624 |
| Right vertical occipital fasciculus | 0.407 ± 0.021 | 0.401 ± 0.028 | 0.578 |

DA, Dopamine agonists; FA, Fractional anisotropy; SD, standard deviation

* The p values were obtained using a one-way analysis of covariance (ANCOVA) with the adjustment for age and sex covariance. Significance level when Bonferroni correction is applied: p < 0.05/42 = 0.0012.
